# Associations between clozapine availability, the diagnosis of treatment-resistant schizophrenia subgroups, antipsychotic monotherapy, and concomitant psychotropics among patients with schizophrenia: a real-world nationwide study

**DOI:** 10.1093/ijnp/pyaf011

**Published:** 2025-03-28

**Authors:** Shinichiro Ochi, Fumitoshi Kodaka, Naomi Hasegawa, Takashi Tsuboi, Kazutaka Ohi, Shun Igarashi, Kentaro Fukumoto, Jun-ichi Iga, Hiroyuki Muraoka, Hitoshi Iida, Hiromi Tagata, Hiroko Kashiwagi, Shusuke Numata, Hirotaka Yamagata, Masahiro Takeshima, Kayo Ichihashi, Naoki Hashimoto, Tatsuya Nagasawa, Toshinori Nakamura, Junya Matsumoto, Hisashi Yamada, Hikaru Hori, Shu-ichi Ueno, Ken Inada, Ryota Hashimoto, Norio Yasui-Furukori

**Affiliations:** Department of Neuropsychiatry, Molecules and Function, Ehime University Graduate School of Medicine, Ehime, Japan; Department of Psychiatry, The Jikei University School of Medicine, Tokyo, Japan; Department of Pathology of Mental Diseases, National Institute of Mental Health, National Center of Neurology and Psychiatry, Tokyo, Japan; Department of Neuropsychiatry, Kyorin University School of Medicine, Tokyo, Japan; Department of Psychiatry, Gifu University Graduate School of Medicine, Gifu, Japan; Department of Pathology of Mental Diseases, National Institute of Mental Health, National Center of Neurology and Psychiatry, Tokyo, Japan; Department of Neuropsychiatry, Kyorin University School of Medicine, Tokyo, Japan; Department of Psychiatry, National Center Hospital, National Center of Neurology and Psychiatry, Tokyo, Japan; Department of Neuropsychiatry, School of Medicine, Iwate Medical University, Iwate, Japan; Department of Neuropsychiatry, Molecules and Function, Ehime University Graduate School of Medicine, Ehime, Japan; Department of Psychiatry, Kitasato University School of Medicine, Kanagawa, Japan; Department of Psychiatry, Faculty of Medicine, Fukuoka University, Fukuoka, Japan; Department of Neuropsychiatry, Toho University Faculty of Medicine, Tokyo, Japan; Department of Forensic Psychiatry, National Center Hospital, National Center of Neurology and Psychiatry, Tokyo, Japan; Department of Psychiatry, Graduate School of Biomedical Science, Tokushima University, Tokushima, Japan; Department of Pathology of Mental Diseases, National Institute of Mental Health, National Center of Neurology and Psychiatry, Tokyo, Japan; Kokoro Hospital Machida, Tokyo, Japan; Department of Neuropsychiatry, Akita University Graduate School of Medicine, Akita, Japan; Department of Neuropsychiatry, University of Tokyo Hospital, Tokyo, Japan; Department of Psychiatry, Hokkaido University Graduate School of Medicine, Hokkaido, Japan; Department of Neuropsychiatry, Kanazawa Medical University, Ishikawa, Japan; Department of Psychiatry, Shinshu University School of Medicine, Nagano, Japan; Department of Pathology of Mental Diseases, National Institute of Mental Health, National Center of Neurology and Psychiatry, Tokyo, Japan; Department of Pathology of Mental Diseases, National Institute of Mental Health, National Center of Neurology and Psychiatry, Tokyo, Japan; Department of Neuropsychiatry, Hyogo Medical University, Hyogo, Japan; Department of Psychiatry, Faculty of Medicine, Fukuoka University, Fukuoka, Japan; Department of Neuropsychiatry, Molecules and Function, Ehime University Graduate School of Medicine, Ehime, Japan; Department of Psychiatry, Kitasato University School of Medicine, Kanagawa, Japan; Department of Pathology of Mental Diseases, National Institute of Mental Health, National Center of Neurology and Psychiatry, Tokyo, Japan; Department of Psychiatry, Dokkyo Medical University School of Medicine, Tochigi, Japan

**Keywords:** implementation strategy, treatment-resistant schizophrenia, polypharmacy, guidelines, EGUIDE

## Abstract

**Background and hypothesis:**

The rate of antipsychotic polypharmacy is high. One risk factor for antipsychotic polypharmacy may be the severity of schizophrenia, including treatment-resistant schizophrenia (TRS). We hypothesized that the institutions that are able to prescribe clozapine present differences in pharmacological treatment even before TRS is diagnosed.

**Study Design:**

A total of 8155 patients with schizophrenia were divided into the clozapine-available institution (CAI) group and the clozapine-unavailable institution (CUI) group. The psychotropic prescription rates at discharge were compared between the two groups. Furthermore, to investigate whether the diagnosis of TRS subgroups influenced treatment efficacy, we compared CAIs and CUIs with descriptions of subgroups with TRS (DSTRS) and those without descriptions of subgroups with TRS (NDSTRS).

**Results:**

Compared to the CUI group, the rates of both antipsychotic monotherapy (58.3% vs. 50.7%; *P* = 2.4 × 10^−7^) and antipsychotic monotherapy without the concomitant use of other psychotropics (20.4% vs. 15.6%; *P* = 3.8 × 10^−5^) were significantly higher in the CAI group. The rate of antipsychotic monotherapy in the CAI with DSTRS group (63.3%) was significantly higher than that in the CAI with NDSTRS group (54.5%; *P* = 1.4 × 10^−12^), the CUI with DSTRS group (49.6%; *P* = 4.9 × 10^−9^), and the CUI with NDSTRS group (50.9%; *P* = 2.0 × 10^−8^). The rate of antipsychotic monotherapy without the concomitant use of other psychotropics in the CAI with DSTRS group (22.6%) was also significantly higher than that in the CAI with NDSTRS group (18.7%; *P* = 4.7 × 10^−4^), the CUI with DSTRS group (15.9%; *P* = 5.5 × 10^−4^), and the CUI with NDSTRS group (15.2%; *P* = 8.0 × 10^−5^). There was no significant difference in these rates between the other groups.

**Conclusions:**

Both the availability of clozapine prescriptions and the precise diagnosis of TRS subgroups at discharge can promote the development of an organizational culture that facilitates the treatment of patients with schizophrenia.

**Significance Statement**

The high rate of antipsychotic polypharmacy for the treatment of schizophrenia is a clinical problem. One risk factor for antipsychotic polypharmacy may be the severity of schizophrenia, including treatment-resistant schizophrenia (TRS). This study focused on the differences in psychotropic prescription rates between clozapine-available institutions (CAIs) and clozapine-unavailable institutions (CUIs) even before TRS was diagnosed. Additionally, CAIs and CUIs were divided into 4 groups based on the descriptions of subgroups with TRS. The prescription rates of both antipsychotic monotherapy and antipsychotic monotherapy without the concomitant use of other psychotropics in the CAI group were significantly higher than those in the CUI group, regardless of whether patients with clozapine prescriptions were included. Furthermore, the prescription rates of both antipsychotic monotherapy and antipsychotic monotherapy without the concomitant use of other psychotropics in the CAI group with descriptions of TRS subgroups were also significantly higher than those in the other groups.

## INTRODUCTION

Schizophrenia is a chronic and complex mental disorder that is characterized by both positive and negative symptoms as well as cognitive impairments.^1^ Antipsychotics are known to alleviate the symptoms of schizophrenia,^2^ and most clinical practice guidelines recommend antipsychotic monotherapy as a first-line treatment for schizophrenia.^2-6^ However, antipsychotics are not effective for approximately 30% of patients with schizophrenia.^7^ Patients who do not respond to at least two different antipsychotics are considered to have treatment-resistant schizophrenia (TRS)^7,8^

Clozapine is the only antipsychotic that is approved for the treatment of TRS; this drug has different properties than other antipsychotics.^9,10^ Compared with most other antipsychotics, clozapine treatment has been shown to have superior efficacy for treating schizophrenia, including TRS.^11-13^ Thus, most clinical practice guidelines recommend clozapine treatment as a first-line treatment for TRS.^2-6^ However, the global prescription rate of clozapine is still low^14-16^ because there are many barriers to using clozapine to treat TRS.^17-19^ For example, clozapine is associated with a greater risk of many other adverse effects, such as neutropenia, pneumonia, myocarditis, seizures, glucose intolerance, and weight gain.^13,20,21^ Therefore, in many countries, the Clozaril Patient Monitoring System (CPMS) registers and monitors patients treated with clozapine and aims to provide countermeasures against these adverse effects, especially neutropenia. In Japan, the CPMS is relatively strict compared with that in other countries. For example, in addition to patients who are prescribed clozapine, all hospitals, psychiatrists, pharmacists, and case administrators must be registered in the CPMS. Furthermore, only when mandatory blood tests are performed and patients are monitored at mandatory intervals (ranging from 7 days until 26 weeks after the initiation of clozapine, followed by biweekly monitoring thereafter) can psychiatrists prescribe clozapine to patients.^22,23^ In June 2021, due to the COVID-19 pandemic, the restriction on dosing after 52 weeks was conditionally reduced to 1 month. Therefore, many hospitals are still unable to prescribe clozapine in Japan.

On the other hand, antipsychotic polypharmacy can increase the risk of many adverse effects, such as extrapyramidal symptoms (EPSs).^2,5^ Thus, many clinical guidelines do not recommend antipsychotic polypharmacy.^2-6^ However, there is still a high rate of antipsychotic polypharmacy in many countries.^24-26^ Although the causes of these differences in rates of polypharmacy among countries are not well known, antipsychotic polypharmacy has been shown to be associated with the severity of schizophrenia.^27^ However, clozapine has been shown to be associated with a higher rate of antipsychotic monotherapy and a lower rate of other concomitant psychotropics.^28^ Thus, the appropriate use of clozapine to treat TRS in accordance with clinical guidelines may contribute to a reduction in antipsychotic polypharmacy.

In Japan, to disseminate guidelines for the treatment of schizophrenia and major depressive disorder through educational programs, we initiated the Effectiveness of Guidelines for Dissemination and Education (EGUIDE) Psychiatric Treatment Project in 2016. Furthermore, to determine the effects of our educational programs, we investigated real-world prescribing patterns and treatment styles associated with nonadherence to the guidelines by assessing prescription rates at discharge for inpatients with schizophrenia.^28-34^ We previously reported differences in the rates of antipsychotic and anticholinergic use in patients with schizophrenia among different institutions,^35,36^ although the detailed causes of these differences among institutions are not well known. Recent research has revealed that one risk factor for adhering to guidelines is organizational culture as an implementation strategy.^37^ Promoting revisions of procedures, protocols, and tasks could lead to the implementation of interventions for organizational culture.^38^ Furthermore, we reported that the rate of TRS diagnosis at various institutions was associated with the rate of clozapine prescription.^39^ This finding suggested that it would be important for interventions in organizational culture to examine the rates of TRS at each institution and the appropriate use of clozapine prescriptions.

However, to our knowledge, no previous studies have examined the association between the appropriate implementation of clozapine and the rate of antipsychotic polypharmacy before patients were diagnosed with TRS. Furthermore, no previous studies examine the effect of the availability of clozapine prescriptions on the overall treatment of patients with schizophrenia across institutions. Therefore, we hypothesized that the institutions that are able to prescribe clozapine would have already presented differences in pharmacological treatment even before a patient developed TRS. We examined the characteristics of psychotropic prescriptions, including antipsychotics, and diagnoses at the time of discharge, depending on whether the institutions were able to prescribe clozapine.

## METHODS

### Patients

This nationwide cross-sectional study was performed as a part of the EGUIDE project. We conducted this study in accordance with the Declaration of Helsinki, and we recruited psychiatrists as participants beginning in October 2016. After the chief researcher at each institution fully explained the study to the participants, we obtained written informed consent from all participants. We collected the medical records of patients at each institution with opt-out consent. We obtained ethical approval from the ethics committees of the National Center of Neurology and Psychiatry (B2022-004) and each participating university/hospital/clinic, and we registered the study protocol in the University Hospital Medical Information Network registry (UMIN000022645).

As we previously reported, we collected data from April to September each year from 2016 to 2020 across 240 institutions.^31^ As we previously reported,^28,29,40^ we collected the following information from each participating institution’s medical records: age, sex, all types and doses of prescribed psychotropics (including antipsychotics, antidepressants, anticholinergic drugs, hypnotics, anxiolytics, mood stabilizers/antiepileptics, and other types of drugs) at both admission and discharge, ECT during each hospitalization, and diagnosis at discharge.

We analyzed data from 11 274 patients from 213 institutions. All patients were diagnosed with schizophrenia at discharge according to the Diagnostic and Statistical Manual of Mental Disorders, fifth edition (DSM-5)^41^ We included patients who were prescribed at least 1 antipsychotic at discharge during their first hospitalization. Furthermore, we excluded all the data from 2016 because we recorded the diagnosis of a subgroup of patients with schizophrenia at discharge, including TRS, non-TRS, suspected TRS, and no description of the diagnosis of the subgroup, in 2017. Ultimately, we analyzed a total of 8155 patients from 207 institutions (44 university hospitals, 45 general hospitals, and 118 psychiatric hospitals).

### Procedure

First, we divided patients into a clozapine-available institution (CAI) group and a clozapine-unavailable institution (CUI) group for comparison.

We compared the mean number of types of prescribed antipsychotics and the mean number of types of prescribed psychotropics, including antipsychotics. Additionally, we compared the rate of “antipsychotic monotherapy,” defined as the prescription of a single antipsychotic regardless of concomitant use of other psychotropics, and the rate of “complete antipsychotic monotherapy,” defined as the prescription of a single antipsychotic without the concomitant use of other psychotropics. Additionally, we compared the prescription rates of other psychotropics, such as anticholinergic drugs, antidepressants, anxiolytics, and hypnotics, and each mood stabilizer. In this study, we converted the doses of each psychotropic drug into chlorpromazine equivalents (CP) for antipsychotics, imipramine equivalents (IP) for antidepressants, biperiden equivalents (BP) for anticholinergics, and diazepam equivalents (DP) for anxiolytics and sleep medications.^42-46^ This approach enabled us to compare the total amount of each psychotropic drug prescribed to patients. However, since the equivalents of ramelteon, hydroxyzine, triclofos sodium, suvorexant, and lemborexant have not been published to date, they were excluded from the comparison of prescription doses. Furthermore, as vortioxetine was not prescribed to any of the patients, there was no need to consider its IP.

We also compared the CAI and CUI groups in terms of institutional attributes, such as university hospitals, general hospitals, and psychiatric hospitals.

Additionally, because we previously reported that clozapine treatment was associated with a higher rate of antipsychotic monotherapy and complete antipsychotic monotherapy,^28^ to avoid the influence of clozapine treatment, we excluded all patients who were prescribed clozapine (*n* = 448) and then compared the 2 groups via the same method.

Moreover, because we previously reported that the rate of clozapine prescription was correlated with the incidence of TRS,^39^ descriptions of TRS or non-TRS as a subgroup diagnosis may influence treatment at the institution. On the basis of the descriptions of the TRS subgroups or no descriptions of the TRS subgroups, we divided the patients into 4 groups: the CAI group with a description of TRS subgroups; the CAI group without a description of TRS subgroups; the CUI group with a description of TRS subgroups; and the CUI group with no description of TRS subgroups. We then compared these 4 groups via the same method.

### Statistical Analysis

Statistical analysis was performed with SPSS 22.0 software (IBM Co.) and EZR 1.54 software (Saitama Medical Center, Jichi Medical University), which is a graphical user interface for R (The R Foundation for Statistical Computing). We also used a modified version of the R commander designed to add statistical functions for biostatistics.^47^ The Shapiro–Wilk test was used to examine the normality of the data. The chi-square test was used to compare categorical variables between the CAI and CUI groups, and the Mann–Whitney *U* test was used to compare continuous and ordered variables between groups. Fisher’s exact test was subsequently used to compare categorical variables among the 4 groups, whereas the Kruskal–Wallis test was used to compare continuous and ordered variables among the 4 groups. We reported *P*-values adjusted by Bonferroni correction for post hoc analyses for multiple comparisons of groups. We defined *P* < 1.9 × 10^−3^ (0.05/27) as significant according to post hoc analyses for multiple comparisons of categories. Descriptive statistics are expressed as the mean ± standard deviation.

## RESULTS

### Differences Between Clozapine-Available Institutions and Clozapine-Unavailable Institutions

There were 6793 patients in the CAI group and 1362 in the CUI group. The characteristics of the patients and the prescription rates of psychotropics at discharge for each group are shown in Table 1.

**Table 1.** Characteristics of patients and prescription rates of psychotropics at discharge at clozapine-available institutions and clozapine-unavailable institutions.

Variables	CAI	CUI	*P-*value
*N*	6793	1362	
Female (%)	3760 (55.4)	727 (53.4)	1.8 × 10^−1^
Age (year)	45.8 (15.6)	48.1 (15.5)	1.3 × 10^−5^*
Descriptions whether TRS or non-TRS (%)	2961 (43.6)	673 (49.4)	7.9 × 10^−5^*
TRS (*N*) (%)	799 (11.8)	113 (8.3)	2.1 × 10^−4^*
Electroconvulsive therapy during hospitalization (%)	356 (5.2)	60 (4.4)	2.0 × 10^−1^
Prescription rate of clozapine (%)	448 (7.0)	0	NA
Mean dose of clozapine (mg/day)	354.5 (158.5)	0	NA
Mean numbers of all types of antipsychotics (*N*/day)	1.5 (0.7)	1.6 (0.7)	1.5 × 10^−7^*
Mean numbers of all types of psychotropics (*N*/day)	3.2 (1.8)	3.5 (1.9)	1.1 × 10^−10^*
Prescription rate of anticholinergic drugs (%)	1726 (25.4)	418 (30.7)	6.8 × 10^−5^*
Prescription rate of antidepressants (%)	538 (7.9)	133 (9.8)	2.4 × 10^−2^
Prescription rate of anxiolytic and hypnotics (%)	4222 (62.2)	939 (68.9)	2.0 × 10^−6^*
Prescription rate of mood stabilizers (%)	1562 (23.0)	359 (26.4)	7.6 × 10^−3^
Prescription rate of valproate (%)	1024 (15.1)	264 (19.4)	6.9 × 10^−5^*
Prescription rate of lithium (%)	447 (6.5)	78 (5.7)	2.4 × 10^−1^
Prescription rate of carbamazepine (%)	179 (2.6)	50 (3.6)	3.5 × 10^−2^
Prescription rate of lamotrigine (%)	63 (0.9)	10 (0.7)	4.9 × 10^−1^

Abbreviations: CAI, clozapine-available institution; CUI, clozapine-unavailable institution; NA, not applicable; TRS, treatment-resistant schizophrenia.

Values are expressed as the mean (SD) except for (%).

^*^ *P* < 1.9 × 10^−3^ was defined as significant.

**Figure 1.**
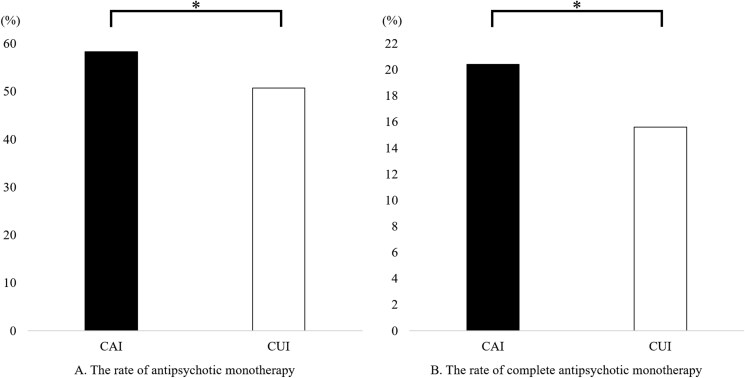
The rates of antipsychotic monotherapy and complete antipsychotic monotherapy between the clozapine-available institution (CAI) group and the clozapine-unavailable institution (CUI) group. (A) The rate of antipsychotic monotherapy in the CAI group was significantly higher than that in the CUI group. (B) The rate of complete antipsychotic monotherapy, defined as antipsychotic monotherapy without concomitant psychotropics, was significantly higher in the CAI group than in the CUI group. The Mann‒Whitney *U* test was used. *P < 1.9 × 10^−3^ was defined as significant.

The rate of antipsychotic monotherapy in the CAI group was significantly higher than that in the CUI group (58.3% vs. 50.7%; *P* = 2.4 × 10^−7^), and the rate of complete antipsychotic monotherapy in the CAI group was significantly higher than that in the CUI group (20.4% vs. 15.6%; *P* = 3.8 × 10^−5^) (Figure 1).

The rate of the description of TRS subgroups was significantly lower in the CAI group than in the CUI group (43.6% vs. 49.4%; *P* = 7.9 × 10^−5^); however, the rate of TRS was significantly higher in the CAI group than in the CUI group (11.8% vs. 8.3%; *P* = 2.1 × 10^−4^).

The prescription rates for some kinds of psychotropics, such as anticholinergic drugs, anxiolytics, hypnotics, and valproate, were significantly lower in the CAI group than in the CUI group. There was no significant difference in the mean dose of each psychotropic agent between the CAI and CUI groups (Table S1).

### Differences Between Clozapine-Available Institutions and Clozapine-Unavailable Institutions When Excluding Patients Who Were Prescribed Clozapine

After excluding patients who were prescribed clozapine, 6345 patients were included in the CAI group, and 1362 patients were included in the CUI group. The characteristics and prescription rates of psychotropics at discharge for each group are shown in Table S2.

The percentage of TRS subgroup descriptions was significantly lower in the CAI group than in the CUI group (40.7% vs. 49.4%; *P* = 3.6 × 10^−9^).

**Figure 2.**
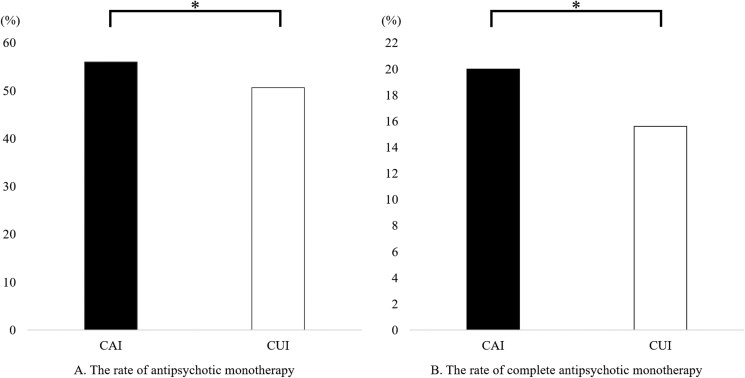
The rates of antipsychotic monotherapy and complete antipsychotic monotherapy in the clozapine-available institution (CAI) group and the clozapine-unavailable institution (CUI) group when patients who were prescribed clozapine were excluded. (A) The rate of antipsychotic monotherapy in the CAI group was significantly higher than that in the CUI group. (B) The rate of complete antipsychotic monotherapy, defined as antipsychotic monotherapy without concomitant psychotropics, was significantly higher in the CAI group than in the CUI group. The Mann‒Whitney *U* test was used. *P < 1.9 × 10^−3^ was defined as significant.

The rate of antipsychotic monotherapy in the CAI group was significantly higher than that in the CUI group (55.9% vs. 50.7%; *P* = 4.0 × 10^−4^), and the rate of complete antipsychotic monotherapy in the CAI group was significantly higher than that in the CUI group (20.0% vs. 15.6%; *P* = 1.8 × 10^−4^) (Figure 2).

The prescription rates of some kinds of psychotropics, such as anticholinergic drugs, anxiolytics, hypnotics, mood stabilizers, and valproate, were significantly lower in the CAI group than in the CUI group.

### Characteristics of the Clozapine-Available and Clozapine-Unavailable Institutions With and Without a Description of TRS Subgroups

The characteristics and prescription rates of psychotropics at discharge for all four groups are shown in Table 2.

**Table 2.** Characteristics of patients and prescription rates of psychotropics at discharge at clozapine-available institutions and clozapine-unavailable institutions, with or without the diagnosis of treatment-resistant schizophrenia subgroups.

Variables	CAI		CUI		*P-*value	CAI + DSTRS vs. CAI + NDSTRS	CAI + DSTRS vs. CUI + DSTRS	CAI + DSTRS vs. CUI + NDSTRS	CAI + NDSTRS vs. CUI + DSTRS	CAI + NDSTRS vs. CUI + NDSTRS	CUI + DSTRS vs. CUI + NDSTRS
	DSTRS	NDSTRS	DSTRS	NDSTRS							
N	2961	3832	673	689							
Female (%)	1671 (56.4)	2089 (54.5)	380 (56.5)	347 (50.4)	2.5 × 10^−2^	6.9 × 10^−1^	1	2.6 × 10^−2^	1	2.8 × 10^−1^	1.6 × 10^−1^
Age (year)	44.8 (15.4)	46.7 (15.7)	47.8 (15.5)	48.3 (15.5)	2.1 × 10^−8^*	7.8 × 10^−5^*	3.8 × 10^−4^*	4.2 × 10^−6^*	8.0 × 10^−1^	8.7 × 10^−2^	1
Electroconvulsive therapy during hospitalization (%)	197 (6.7)	159 (4.1)	40 (5.9)	20 (2.9)	1.4 × 10^−6^*	3.7 × 10^−5^*	1	4.4 × 10^−4^*	2.5 × 10^−1^	8.2 × 10^−1^	4.7 × 10^−2^
Prescription rate of clozapine (%)	378 (12.8)	70 (1.8)	0	0	NA						
Mean dose of clozapine (mg/day)	351.7 (156.5)	369.8 (169.8)	0	0	NA						
Mean numbers of all types of antipsychotics (N/day)	1.5 (0.7)	1.6 (0.7)	1.6 (0.7)	1.6 (0.7)	3.9 × 10^−16^*	2.7 × 10^−11^*	3.6 × 10^−9^*	4.0 × 10^−8^*	1.6 × 10^−1^	4.8 × 10^−1^	1
Mean numbers of all types of psychotropics (N/day)	3.0 (1.8)	3.3 (1.8)	3.4 (1.7)	3.6 (2.0)	5.8 × 10^−14^*	1.9 × 10^−5^*	1.3 × 10^−6^*	8.0 × 10^−11^*	5.9 × 10^−2^	1.1 × 10^−4^*	9.5 × 10^−1^
Prescription rate of anti-cholinergic drugs (%)	689 (23.3)	1037 (27.1)	190 (28.2)	228 (33.1)	4.0 × 10^−7^*	2.4 × 10^−3^	4.9 × 10^−2^	1.0 × 10^−6^*	1	8.7 × 10^−3^	3.2 × 10^−1^
Prescription rate of antidepressants (%)	243 (8.2)	295 (7.7)	46 (6.8)	87 (12.6)	2.9 × 10^−4^*	1	1	3.0 × 10^−3^	1	3.2 × 10^−4^*	2.1 × 10^−3^
Prescription rate of anxiolytic and hypnotics (%)	1800 (60.8)	2422 (63.2)	468 (69.5)	471 (68.4)	5.4 × 10^−6^*	2.6 × 10^−1^	1.4 × 10^−4^*	1.3 × 10^−3^*	8.8 × 10^−3^	5.8 × 10^−2^	1
Prescription rate of mood stabilizers (%)	689 (23.3)	873 (22.8)	160 (23.8)	199 (28.9)	7.7 × 10^−3^	1	1	1.3 × 10^−2^	1	3.9 × 10^−3^	2.2 × 10^−1^
Prescription rate of valproate (%)	424 (14.3)	600 (15.7)	113 (16.8)	151 (21.9)	3.2 × 10^−5^*	7.9 × 10^−1^	6.2 × 10^−1^	1.1 × 10^−5^*	1	4.6 × 10^−4^*	1.2 × 10^−1^
Prescription rate of lithium (%)	216 (7.3)	231 (6.0)	41 (6.1)	37 (5.4)	1.2 × 10^−1^	2.3 × 10^−1^	1	4.8 × 10^−1^	1	1	1
Prescription rate of carbamazepine (%)	80 (2.7)	99 (2.6)	19 (2.8)	31 (4.5)	6.1 × 10^−2^	1	1	1.1 × 10^−1^	1	5.3 × 10^−2^	6.8 × 10^−1^
Prescription rate of lamotrigine (%)	34 (1.1)	29 (0.8)	1 (0.1)	9 (1.3)	2.4 × 10^−2^	5.9 × 10^−1^	8.3 × 10^−2^	1	4.5 × 10^−1^	1	1.3 × 10^−1^

Abbreviations: CAI, clozapine-available institution; CUI, clozapine-unavailable institution; DSTRS, the description of subgroups about treatment-resistant schizophrenia; NA, not applicable; NDSTRS, no description of subgroups about treatment-resistant schizophrenia.

Values are expressed as the mean (SD) except for (%).

*P < 1.9 × 10^−3^ was defined as significant.

**Figure 3.**
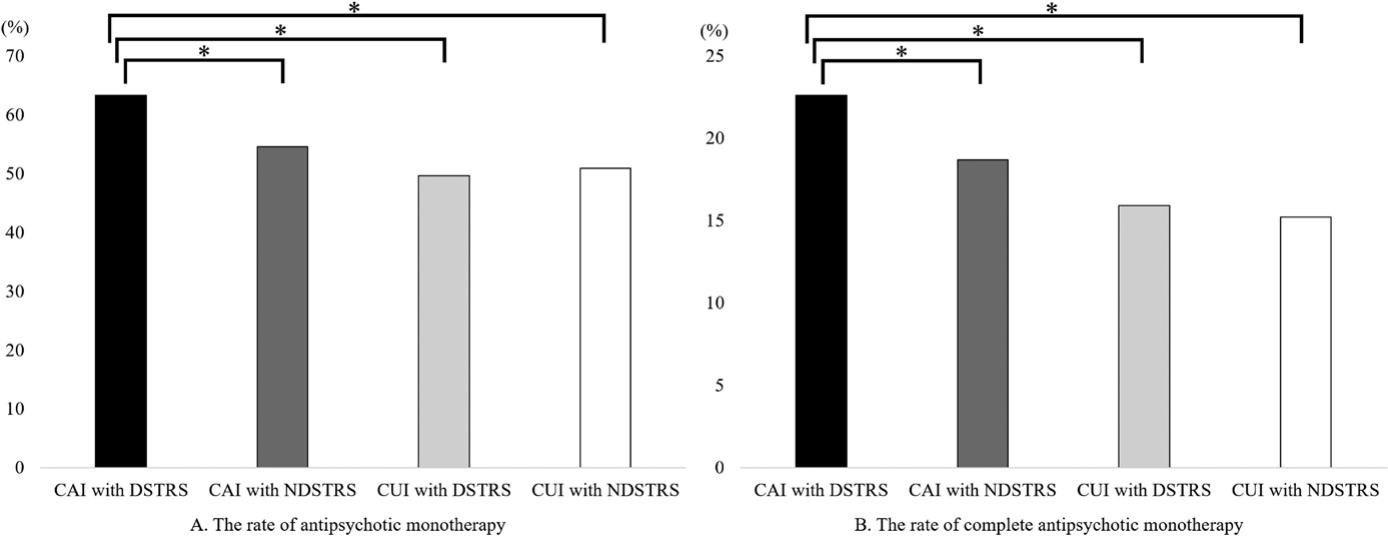
The rates of antipsychotic monotherapy and complete antipsychotic monotherapy between the clozapine-available institution (CAI) group and the clozapine-unavailable institution (CUI) group, regardless of whether treatment-resistant schizophrenia subgroups were described. (A) The rate of antipsychotic monotherapy in the CAI group with a description of treatment-resistant schizophrenia (TRS) subgroups was significantly higher than that in the other groups. (B) The rate of complete antipsychotic monotherapy, defined as antipsychotic monotherapy without concomitant psychotropics, was significantly higher in the CAI group with a description of TRS subgroups, than in the other groups. The Mann‒Whitney *U* test adjusted by Bonferroni correction was used. *P < 1.9 × 10^−3^ was defined as significant. DSTRS, the description of subgroups about treatment-resistant schizophrenia; NDSTRS, no description of subgroups about treatment-resistant schizophrenia.

The rate of antipsychotic monotherapy in the CAI group with a description of TRS subgroups (63.3%) was significantly higher than that in the CAI group without a description of TRS subgroups (54.5%; *P* = 1.4 × 10^−12^), the CUI group with a description of TRS subgroups (49.6%; *P* = 4.9 × 10^−9^), and the CUI group without a description of TRS subgroups (50.9%; *P* = 2.0 × 10^−8^). The rate of complete antipsychotic monotherapy was significantly higher in the CAI group with a description of TRS subgroups (22.6%) than in the CAI group without a description of TRS subgroups (18.7%; *P* = 4.7 × 10^−4^), the CUI group with a description of TRS subgroups (15.9%; *P* = 5.5 × 10^−4^), and the CUI group without a description of TRS subgroups (15.2%; *P* = 8.0 × 10^−5^) (Figure 3).

Additionally, the prescription rate of anxiolytics and hypnotics was significantly lower in the CAI group with a description of TRS subgroups (60.8%) than in the CUI group with a description of TRS subgroups (69.5%; *P* = 1.4 × 10^−4^) and the CUI group without a description of TRS subgroups (68.4%; *P* = 1.3 × 10^−3^). The prescription rates of anticholinergic drugs and valproate were significantly lower in the CAI group with a description of TRS subgroups than in the CUI group without a description of TRS subgroups. There was no significant difference in the mean dose of each psychotropic agent among the 4 groups (Table S3).

## DISCUSSION

In this study, we observed a significantly higher rate of both antipsychotic monotherapy and complete antipsychotic monotherapy in the CAI group than in the CUI group, regardless of whether patients with clozapine prescriptions were included. On the other hand, the prevalence of descriptions of TRS subgroups was lower in the CAI group. Furthermore, we reported that the rates of both antipsychotic monotherapy and complete antipsychotic monotherapy were higher in the CAI group, with descriptions of TRS subgroups, than in the other groups. To our knowledge, this is the first study to reveal that both CAIs and the precise diagnosis of TRS subgroups are associated with increased rates of antipsychotic monotherapy and complete antipsychotic monotherapy.

This result shows that institutional characteristics, especially the availability of clozapine, may influence the rate of antipsychotic monotherapy or complete antipsychotic monotherapy. Although few studies have examined the influence of institutional characteristics on the treatment behavior of clinicians, Kinney et al.^48^ identified the factors influencing the recommended clinical practice guidelines for post-concussion sleep disturbance and headache, including institutional culture, institutional resources, and the interaction between culture and resources.^48^ This finding is consistent with our results. In Japan, with respect to the efficacy of clozapine, TRS is defined as an insufficient response after the administration of two or more antipsychotics at sufficient doses (ie, at least 600 mg/day of CP) for more than 4 weeks, including at least 1 atypical antipsychotic^49^ The treatment for clozapine prescriptions in the future and the correct diagnosis of TRS according to this protocol are almost the same as those recommended by the guidelines,^2,8^ and these recommendations may help to build a treatment culture, which may facilitate the development of future guidelines with respect to rates of antipsychotic monotherapy. Furthermore, we recently reported that an educational program for clinical guidelines was effective in terms of improving the clinical behavior of psychiatrists.^50-52^ These results may support the development of more institutional culture guideline-recommended treatments. The availability of clozapine in institutions is an important resource for the treatment of schizophrenia.

Furthermore, we revealed that the rates of both antipsychotic monotherapy and complete antipsychotic monotherapy were significantly higher in the CAI group with a description of TRS subgroups than in the other groups. A previous review reported that one of the clinical barriers to clozapine use was the difficulty in identifying adequate patients and unclear diagnoses^18^ Furthermore, we previously reported that the prescription rate of clozapine was significantly correlated with the prevalence of TRS, and the prescription rate of clozapine in institutions with a high prevalence of TRS was significantly higher than that in institutions with a low prevalence of TRS.^39^ These results support the findings of the current study, indicating that making a precise diagnosis of TRS or non-TRS for each patient with schizophrenia is important for developing treatment strategies, including clozapine prescriptions. However, the CUI group had a significantly higher prevalence of descriptions of TRS subgroups than did the CAI group; however, the rates of antipsychotic monotherapy and complete antipsychotic monotherapy were lower in the CUI group. Furthermore, while the precise diagnosis of TRS subgroups at discharge was associated with a greater percentage of patients receiving antipsychotic monotherapy in the CAI group, this finding was not apparent in the CUI group. These findings suggest that both the availability of clozapine and a precise diagnosis of TRS may be important for increasing the rates of antipsychotic monotherapy and complete antipsychotic monotherapy due to the interaction between culture and resources.

Recently, the importance of dissemination and implementation strategies has been reported to address the gap between clinical guidelines and daily clinical practice.^37,53-56^ Recent meta-analyses reported that organizational culture alone is a risk factor for adherence to guidelines^38^; this finding is consistent with the results of the present study. The development of guideline-based institutional culture as a result of this study could be applied to other psychiatric problems worldwide as an implementation strategy approach.

This study has several limitations. First, this was a cross-sectional study, and we could not sufficiently clarify the relationships between CAIs and a higher rate of antipsychotic monotherapy or complete antipsychotic monotherapy. Second, sampling bias and selection bias may have affected the results because there are few CAIs in Japan. Furthermore, because we could not assess the quality of psychiatrists who work at each institution, we could not rule out the possibility that psychiatrists who work at CAIs were of higher quality than those working at CUIs; therefore, institutional bias may have affected the results. Third, we did not assess the clinical symptoms or severity of schizophrenia using rating scales such as the Positive and Negative Syndrome Scale or the Brief Psychiatric Rating Scale. However, Kitagawa et al.^57^ recently reported changes in prescription surveys before and after the introduction of clozapine at one institution. They reported an increased rate of antipsychotic monotherapy and a decreased rate of polypharmacy after the introduction of clozapine. Although 1 institution cannot solve the problem of the severity of schizophrenia completely, this finding may support our suggestion of the importance of organizational culture. Fourth, we investigated only prescriptions at discharge and could not investigate subsequent outcomes. Recently, Taipale et al.^58^ reported that compared with antipsychotic monotherapy, antipsychotic polypharmacy was associated with a significantly lower risk of cardiovascular hospitalization and superior safety at high doses. However, previous studies reported that an increase in the number of patients receiving antipsychotics, anticholinergic drugs, and benzodiazepines was associated with an increased risk of neuroleptic malignant syndrome^59^; furthermore, high exposure to benzodiazepines among patients with schizophrenia was significantly associated with mortality, but this finding did not hold for exposure to antipsychotics and antidepressants.^60^ Thus, regarding safety, interactions between multiple antipsychotics as well as interactions between antipsychotics and other psychotropics have yet to be fully elucidated. To enhance knowledge regarding the safety of psychotropics for schizophrenia patients—including knowledge about antipsychotic monotherapy with or without concomitant psychotropic medications, three or more antipsychotic polypharmacy agents, and psychotropic polypharmacy agents—further large cohort studies are needed.

In conclusion, both the establishment of CAIs and the precise diagnosis of TRS subgroups at discharge at each institution could lead to the development of an organizational culture that would facilitate a higher psychotropic monotherapy rate for overall SCZ treatment.

## Supplementary Material

pyaf011_suppl_Supplementary_Table_S1

pyaf011_suppl_Supplementary_Table_S2

pyaf011_suppl_Supplementary_Table_S3

## Data Availability

The data are not publicly available due to privacy and ethical restrictions (ie, we did not obtain informed consent on the public availability of the raw data).
