# Supplementary material for: Associations between clozapine availability, the diagnosis of treatment-resistant schizophrenia subgroups, antipsychotic monotherapy, and concomitant psychotropics among patients with schizophrenia: a real-world nationwide study
Source: Int J Neuropsychopharmacol. 2025 Mar 28;28(4):pyaf011. doi: 10.1093/ijnp/pyaf011 (PMC11986582; doi:10.1093/ijnp/pyaf011)
Supplement: pyaf011_suppl_Supplementary_Table_S1 [file pyaf011_suppl_supplementary_table_s1.docx]

**Supplementary Table 1. Mean dose of psychotropics at discharge at clozapine-available institutions and clozapine-unavailable institutions.**

| Variables | CAI | CUI | *p* value |
| --- | --- | --- | --- |
| N | 6793 | 1362 |  |
| Mean dose of total antipsychotics (mg/day)^†1^ | 691.4 (442.7) | 685.4 (454.0) | 2.9 × 10^-1^ |
| Mean dose of atypical antipsychotics (mg/day)^†1^ | 666.2 (402.5) | 651.7 (409.9) | 5.7 × 10^-2^ |
| Mean dose of typical antipsychotics (mg/day)^†1^ | 276.2 (326.3) | 258.9 (285.5) | 6.7 × 10^-1^ |
| Mean dose of anti-cholinergic drugs (mg/day)^†2^ | 2.6 (1.5) | 2.6 (1.5) | 1 |
| Mean dose of antidepressants (mg/day)^†3^ | 89.7 (80.1) | 80.3 (73.4) | 1.6 × 10^-1^ |
| Mean dose of anxiolytic and hypnotics (mg/day)^†4^ | 13.4 (14.6) | 13.5 (14.2) | 4.1 × 10^-1^ |
| Mean dose of valproate (mg/day) | 647.6 (281.5) | 591.7 (258.1) | 2.8 × 10^-3^ |
| Mean dose of lithium (mg/day) | 577.6 (242.9) | 552.6 (209.3) | 3.8 × 10^-1^ |
| Mean dose of carbamazepine (mg/day) | 440.5 (218.1) | 424.0 (186.1) | 7.9 × 10^-1^ |
| Mean dose of lamotrigine (mg/day) | 161.5 (106.0) | 177.5 (122.7) | 6.4 × 10^-1^ |

The values are expressed as the means (SDs). *···*p* < 1.9 × 10^-3^ was defined as significant. CAI: clozapine-available institution; CUI: clozapine-unavailable institution; TRS: treatment-resistant schizophrenia. †1: presented as chlorpromazine equivalent, †2: presented as biperiden equivalent, †3: presented as imipramine equivalent, *†4: presented as diazepam equivalent.
