# Supplementary material for: Associations between clozapine availability, the diagnosis of treatment-resistant schizophrenia subgroups, antipsychotic monotherapy, and concomitant psychotropics among patients with schizophrenia: a real-world nationwide study
Source: Int J Neuropsychopharmacol. 2025 Mar 28;28(4):pyaf011. doi: 10.1093/ijnp/pyaf011 (PMC11986582; doi:10.1093/ijnp/pyaf011)
Supplement: pyaf011_suppl_Supplementary_Table_S2 [file pyaf011_suppl_supplementary_table_s2.docx]

**Supplementary Table 2. Characteristics of patients and prescription rates of psychotropics at discharge at clozapine-available institutions and clozapine-unavailable institutions when excluding patients who were prescribed clozapine.**

| Variables | CAI | CUI | *p* value |
| --- | --- | --- | --- |
| N | 6345 | 1362 |  |
| Female (%) | 3525 (55.6) | 727 (53.4) | 1.4 × 10^-1^ |
| Age (year) | 46.2 (15.8) | 48.1 (15.5) | 3.4 × 10^-4*^ |
| Descriptions whether TRS or non-TRS (%) | 2583 (40.7) | 673 (49.4) | 3.6 × 10^-9*^ |
| TRS(N) (%) | 424 (6.7) | 113 (8.3) | 3.9 × 10^-2^ |
| Electroconvulsive therapy during hospitalization (%) | 325 (5.1) | 60 (4.4) | 2.7 × 10^-1^ |
| Mean numbers of all types of antipsychotics (N/day) | 1.5 (0.7) | 1.6 (0.7) | 2.7 × 10^-4*^ |
| Mean numbers of all types of psychotropics (N/day) | 3.2 (1.8) | 3.5 (1.9) | 3.9 × 10^-8*^ |
| Prescription rate of anti-cholinergic drugs (%) | 1674 (26.4) | 418 (30.7) | 1.4 × 10^-3*^ |
| Prescription rate of antidepressants (%) | 515 (8.1) | 133 (9.8) | 4.7 × 10^-2^ |
| Prescription rate of anxiolytic and hypnotics (%) | 3994 (62.9) | 939 (68.9) | 2.9 × 10^-5*^ |
| Prescription rate of mood stabilizers (%) | 1388 (21.9) | 359 (26.4) | 3.4 × 10^-4*^ |
| Prescription rate of valproate (%) | 975 (15.4) | 264 (19.4) | 2.5 × 10^-4*^ |
| Prescription rate of lithium (%) | 324 (5.1) | 78 (5.7) | 3.5 × 10^-1^ |
| Prescription rate of carbamazepine (%) | 176 (2.8) | 50 (3.6) | 7.5 × 10^-2^ |
| Prescription rate of lamotrigine (%) | 50 (0.8) | 10 (0.7) | 8.4 × 10^-1^ |
| Mean dose of total antipsychotics (mg/day)^†1^ | 687.6 (446.9) | 685.4 (454.0) | 5.7 × 10^-1^ |
| Mean dose of atypical antipsychotics (mg/day)^†1^ | 660.8 (405.9) | 651.7 (409.9) | 2.1 × 10^-1^ |
| Mean dose of typical antipsychotics (mg/day)^†1^ | 276.0 (321.9) | 258.9 (285.5) | 6.2 × 10^-1^ |
| Mean dose of anti-cholinergic drugs (mg/day)^†2^ | 2.6 (1.5) | 2.6 (1.5) | 9.8 × 10^-1^ |
| Mean dose of antidepressants (mg/day)^†3^ | 89.7 (80.1) | 80.3 (73.4) | 1.5 × 10^-1^ |
| Mean dose of anxiolytic and hypnotics (mg/day)^†4^ | 13.4 (14.7) | 13.5 (14.2) | 4.7 × 10^-1^ |
| Mean dose of valproate (mg/day) | 645.3 (280.9) | 591.7 (258.1) | 4.2 × 10^-3^ |
| Mean dose of lithium (mg/day) | 608.0 (239.6) | 552.6 (209.3) | 4.7 × 10^-2^ |
| Mean dose of carbamazepine (mg/day) | 441.5 (217.0) | 424.0 (186.1) | 7.8 × 10^-1^ |
| Mean dose of lamotrigine (mg/day) | 156.5 (105.7) | 177.5 (122.7) | 5.3 × 10^-1^ |

Values are expressed as the mean (SD) except for (%). *···*p* < 1.9 × 10^-3^ was defined as significant. CAI: clozapine-available institution; CUI: clozapine-unavailable institution; TRS: treatment-resistant schizophrenia. †1: presented as chlorpromazine equivalent, †2: presented as biperiden equivalent, †3: presented as imipramine equivalent, *†4: presented as diazepam equivalent.
