# Supplementary material for: Associations between clozapine availability, the diagnosis of treatment-resistant schizophrenia subgroups, antipsychotic monotherapy, and concomitant psychotropics among patients with schizophrenia: a real-world nationwide study
Source: Int J Neuropsychopharmacol. 2025 Mar 28;28(4):pyaf011. doi: 10.1093/ijnp/pyaf011 (PMC11986582; doi:10.1093/ijnp/pyaf011)
Supplement: pyaf011_suppl_Supplementary_Table_S3 [file pyaf011_suppl_supplementary_table_s3.docx]

**Supplementary Table 3. Mean dose of psychotropics at discharge at clozapine-available institutions and clozapine-unavailable institutions, with or without the diagnosis of treatment-resistant schizophrenia subgroups.**

| Variables | CAI | |  | CUI | | *p* value |
| --- | --- | --- | --- | --- | --- | --- |
|  | DSTRS | NDSTRS |  | DSTRS | NDSTRS |  |
| N | 2961 | 3832 |  | 673 | 689 |  |
| Mean dose of total antipsychotics (mg/day)^†1^ | 689.9 (426.5) | 692.5 (454.8) |  | 689.4 (446.1) | 681.4 (461.8) | 5.5 × 10^-1^ |
| Mean dose of atypical antipsychotics (mg/day)^†1^ | 665.0 (386.7) | 667.2 (414.7) |  | 649.8 (402.3) | 653.6 (417.8) | 2.5 × 10^-1^ |
| Mean dose of typical antipsychotics (mg/day)^†1^ | 279.9 (341.4) | 274.2 (317.7) |  | 242.7 (246.6) | 273.9 (317.1) | 8.7 × 10^-1^ |
| Mean dose of anti-cholinergic drugs (mg/day)^†2^ | 2.7 (1.5) | 2.6 (1.6) |  | 2.6 (1.5) | 2.6 (1.5) | 7.4 × 10^-1^ |
| Mean dose of antidepressants (mg/day)^†3^ | 88.2 (77.5) | 90.9 (82.2) |  | 82.8 (81.8) | 79.0 (69.0) | 5.5 × 10^-1^ |
| Mean dose of anxiolytic and hypnotics (mg/day)^†4^ | 12.8 (13.6) | 13.8 (15.2) |  | 12.7 (12.5) | 14.3 (15.6) | 5.7 × 10^-2^ |
| Mean dose of valproate (mg/day) | 645.8 (286.6) | 648.8 (278.1) |  | 594.7 (251.7) | 589.5 (263.6) | 2.7 × 10^-2^ |
| Mean dose of lithium (mg/day) | 547.7 (233.5) | 605.6 (248.7) |  | 543.9 (218.0) | 562.2 (201.9) | 5.5 × 10^-2^ |
| Mean dose of carbamazepine (mg/day) | 414.4 (196.8) | 461.6 (232.6) |  | 381.6 (188.0) | 450.0 (183.0) | 3.2 × 10^-1^ |
| Mean dose of lamotrigine (mg/day) | 171.3 (82.1) | 150.0 (129.0) |  | 200.0 | 175.0 (129.9) | 5.9 × 10^-1^ |

The values are expressed as the means (SDs). *···*p* < 1.9 × 10^-3^ was defined as significant. CAI: clozapine-available institution; CUI: clozapine-unavailable institution; DSTRS: the description of subgroups about treatment-resistant schizophrenia; NDSTRS: no description of subgroups about treatment-resistant schizophrenia. †1: presented as chlorpromazine equivalents, †2: presented as biperiden equivalents, †3: presented as imipramine equivalents, †4: presented as diazepam equivalents.
